# Royal Hospital for Diseases of the Chest, City Road

**Published:** 1902-08-16

**Authors:** 


### Around the Hospitals.

### ROYAL HOSPITAL FOR DISEASES OF THE CHEST, CITY ROAD.

THE YEAR'S WORK.

The number of patients under treatment during 1901 was —in-patients, 709; out-patients, 6,875 new cases; 3,865 renewed; total attendances, 26,884. Of the in-patients, 561 were discharged convalescent or relieved, 78 died, and 70 remained at the close of the year. The total ordinary income was £9,134; extraordinary, £522 10s. There was a loss of £85 on annual subscriptions, but donations increased by £2,543. The ordinary expenditure was £8,400; extraordinary, £2,085 13s. 4d. The Hospital Sunday Fund gave £333 6s. 8d.; Hospital Saturday Fund, £156 15s., and King Edward's Fund, £550. A site has been purchased for the much-needed nurses' home.

